# Dead Shrimp Blues: A Global Assessment of Extinction Risk in Freshwater Shrimps (Crustacea: Decapoda: Caridea)

**DOI:** 10.1371/journal.pone.0120198

**Published:** 2015-03-25

**Authors:** Sammy De Grave, Kevin G. Smith, Nils A. Adeler, Dave J. Allen, Fernando Alvarez, Arthur Anker, Yixiong Cai, Savrina F. Carrizo, Werner Klotz, Fernando L. Mantelatto, Timothy J. Page, Jhy-Yun Shy, José Luis Villalobos, Daisy Wowor

**Affiliations:** 1 Oxford University Museum of Natural History, Oxford, United Kingdom; 2 Global Species Programme, International Union for Conservation of Nature, Cambridge, United Kingdom; 3 Colección Nacional de Crustáceos, Instituto de Biología, Universidad Nacional Autónoma de México, México City, México; 4 Tropical Marine Science Institute, National University of Singapore, Singapore; 5 National Parks Board, Singapore; 6 Private Researcher, Rum, Austria; 7 Laboratory of Bioecology and Crustacean Systematics, Department of Biology, University of São Paulo, Ribeirão Preto, Brazil; 8 Australian Rivers Institute, Griffith University, Nathan, Australia; 9 National Penghu University of Science and Technology, Penghu, Taiwan; 10 Division of Zoology, Research Center for Biology, Indonesian Institute of Sciences (LIPI), Cibinong, Indonesia; Shanghai Ocean University, CHINA

## Abstract

We present the first global assessment of extinction risk for a major group of freshwater invertebrates, caridean shrimps. The risk of extinction for all 763 species was assessed using the IUCN Red List criteria that include geographic ranges, habitats, ecology and past and present threats. The Indo-Malayan region holds over half of global species diversity, with a peak in Indo-China and southern China. Shrimps primarily inhabit flowing water; however, a significant subterranean component is present, which is more threatened than the surface fauna. Two species are extinct with a further 10 possibly extinct, and almost one third of species are either threatened or Near Threatened (NT). Threats to freshwater shrimps include agricultural and urban pollution impact over two-thirds of threatened and NT species. Invasive species and climate change have the greatest overall impact of all threats (based on combined timing, scope and severity of threats).

## Introduction

Freshwater habitats occupy less than 1% of the Earth’s surface [1], but harbour nearly 10% of the world’s species [2], 85% of which are invertebrates [3]. Inland waters also provide important ecosystem services, which are typically more valuable than in other ecosystem types, and are often critical in supporting the livelihoods of the poorest communities [4]. However, the world has lost approximately 71% of its wetlands since 1900 [5], due to wide scale threats and undervaluation by decision makers, and freshwater species have declined by an average of 76% since 1970 [6], making freshwater habitats one of the most imperilled across the globe [2, 7]. There is an urgent need for policies and management governing freshwater systems to be better informed (CBD SBSTTA 2010), so that remaining biodiversity is protected and that continued ecosystem service provision is safeguarded, as recognised in the UN Convention on Biological Diversity (CBD) Aichi Targets. Nevertheless, despite decades of studies, a global overview of freshwater biodiversity remains difficult to obtain for many groups [8]. Nowhere is this dearth of information in more need of addressing than in conservation science, with the IUCN’s Red List of Threatened Species containing very few global comprehensive invertebrate assessments, although the majority of vertebrate taxa have been completed [9].

Here we present the first global conservation assessment of freshwater caridean shrimps, based on their distribution, ecology, past and present threats and conservation actions.

**Fig 1:**
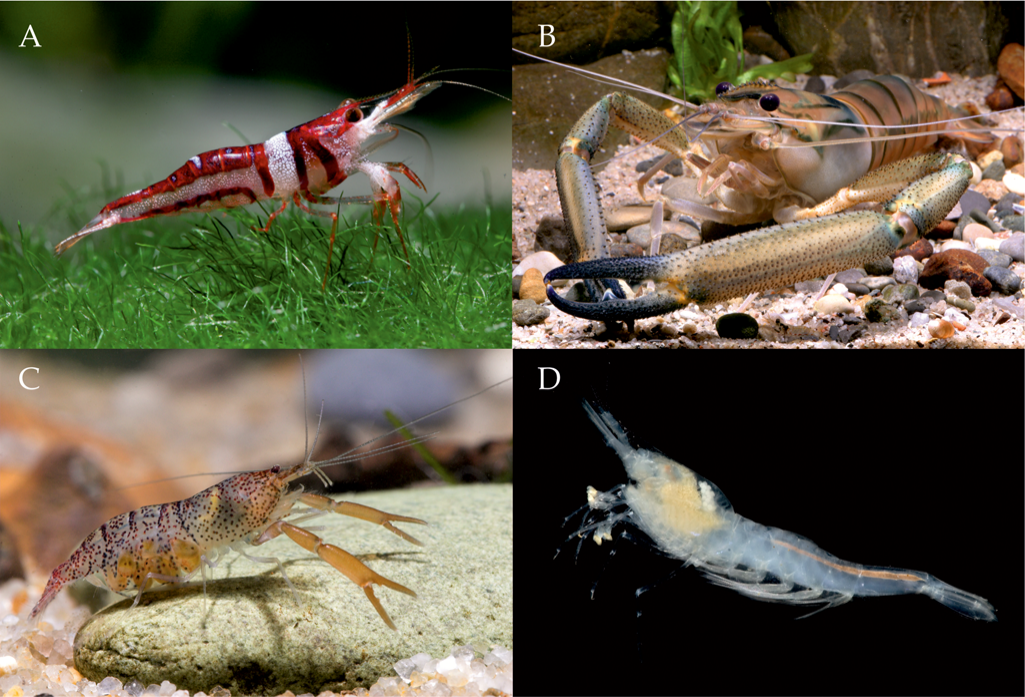
Examples of freshwater shrimps with assessment of their risk of extinction status. (A) *Caridina woltereckae* (**EN**), endemic to Lake Towuti (Sulawesi), currently under threat due to overharvesting for the aquarium trade, pollution and invasive fish species; (B) *Macrobrachium vollenhoveni* (**LC**), a widespread African species, ranging from Senegal to Angola; (C) *Euryrhynchus amazoniensis* (**LC**), a widespread Amazonian species; (D) *Palaemonias alabamae* (**EN**), known from only four cave systems in Alabama, USA, currently under threat from groundwater abstraction and habitat change.

Caridean shrimps (hereafter “shrimps”) (Fig. 1) are a highly species-rich group of decapod crustaceans (Decapoda: Caridea), second only to crabs (Decapoda: Brachyura). Globally, around 3500 species were known in 2011 [10] with a significant number of additions since then. Approximately 770–800 species live in freshwater and related continental waters (e.g. anchialine caves), amounting to about a fifth of global shrimp diversity.

Freshwater shrimps are particularly important from a human perspective in tropical and subtropical freshwater ecosystems where numerous species are wild-harvested or farmed for food [11]. Additionally, global industrial scale production of freshwater shrimps is in excess of 440 000 tonnes per annum with a total value of US$ 2.2 billion [12], and in contrast to most other freshwater invertebrate groups, an increasing number of species are being made available in the aquarium trade. Relatively few freshwater groups inhabit the full range of environmental conditions as do shrimps, which dwell in slow flowing rivers near the sea, large ancient lakes, swamps, small streams and montane lakes, as well as in varied subterranean habitats. Taken as a group, their broad species level ecologies are rather unique within freshwater invertebrates [13], from filter feeders to micro-predators, as well as differential life-histories, ranging from direct development to seaward migrations,.

Caridean shrimps are thus potentially an excellent, but overlooked, flagship group in freshwater conservation, as they (1) are a significant (sometimes dominant) component of freshwater ecosystems, especially in the tropics; (2) display a range of life-histories with many species migrating towards the sea to release larvae; (3) occur in a wide range of habitats; (4) have clear cultural links, due to artisanal fishing practices; and (5) are taxonomically comprehensively studied, with global species and distribution lists already available [10, 13].

## Methods

Non-marine shrimp taxa were extracted from the global checklist by De Grave and Fransen [10], supplemented by newly described taxa up to March 2013. Freshwater taxa were defined as those permanently residing in freshwater or requiring freshwater to complete their lifecycle. Anchialine species living in the deeper, more saline parts of caves and pools were excluded, as were fully brackish water species. In total 763 species and an additional 38 subspecies were included in the present assessment. Data were submitted for validation to the 2014 Red List update [14]; taxa described since March 2013 are not included in the current assessment.

The risk of extinction for each species was assessed according to IUCN Red List Categories and Criteria version 3.1 [15] with information including geographic range, population, habitat requirements, and threats being collated in order to assign a Red List Category to each species. The categories of extinction risk are Extinct (EX); Extinct in the Wild (EW); Critically Endangered (CR); Endangered (EN); Vulnerable (VU); Near Threatened (NT); Least Concern (LC); and Data Deficient (DD). Those species assessed as CR, EN or VU are termed ‘threatened’. The assessments were then reviewed by independent experts at regional workshops in 2011 and 2012 and through email correspondence. The distribution of each species was mapped to individual sub-basins, as delineated by the HydroBASINS global catchment layer [16] using ArcGIS (ESRI, Redlands, CA). The data and numbers presented herein concern only taxa assessed at species level. All species assessments and maps are available online on the IUCN Red List of Threatened Species (www.iucnredlist.org), where spatial data can also be downloaded.

To help distinguish between major and minor threats to a species, each threat recorded in an IUCN Red List assessment is qualified by the *timing* of the threat (past, ongoing, future, past likely to return); the *scope* of the threat i.e. how much of the population is impacted by the threat (whole >90%, majority 50–90%, minority <50%); and the *severity* of the threat i.e. what is the impact of the threat (very rapid declines, rapid declines, slow significant declines, causing/could cause fluctuations, negligible declines, no declines). The timing, scope and severity are then used to calculate an *impact score* (see www.iucnredlist.org for more information), which is useful for distinguishing between major and minor threats [17].

## Results

**Fig 2:**
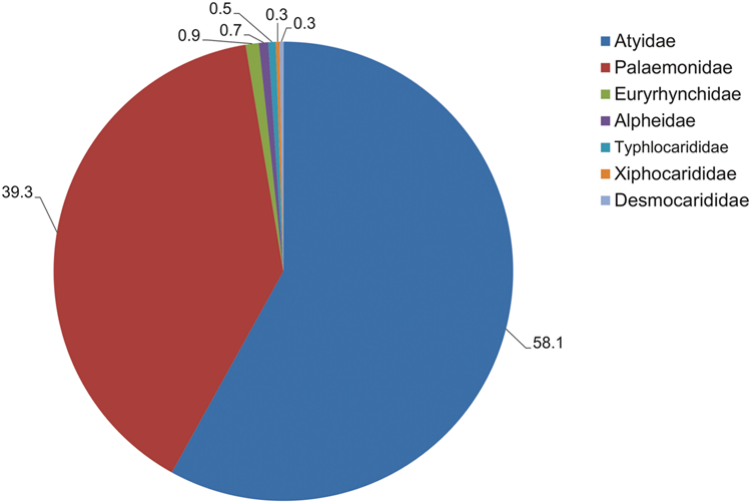
Freshwater shrimp species richness across the constituent families, expressed as a percentage of global fauna.

Freshwater shrimps are only present in seven families out of 38 currently recognised families of Caridea [18]. Of these, two families, Atyidae and Palaemonidae, numerically dominate the fauna, comprising 443 and 300 species respectively, and thus account for 97.4% of all freshwater shrimp species (Fig. 2). Both families occur in all biogeographical realms, except Antarctica, but exhibit their highest diversity in the Indo-Malayan realm [13]. Although as a whole, Atyidae are often considered to be restricted to freshwater, several brackish and anchialine genera are known [13], whilst a marine-based larval dispersal phase, plays a significant role in their distribution [19]. The family comprises of in excess of 40 genera, but is numerically dominated by the genus *Caridina* (Fig. 1A), which comprises 290 species [10], of which 280 were included in the present assessment. Over three-quarter of species diversity in the genus occurs in the Indo-Malayan realm, with additional species present in the Australasian, Afrotropical and Oceanian realms and a minor component in the eastern part of the Palearctic realm. In the Nearctic and Neotropical realms, the family is represented by more range-restricted genera, including *Atya*. The Palaemonidae are primarily a marine family, which reaches its peak biodiversity on tropical coral reefs, but also contains the species-rich, freshwater genus, *Macrobrachium* (Fig. 1B), with 243 species [10], of which 239 were included in the present assessment. The global distribution of this genus comprises all realms (except Antarctica), although few species are present in the eastern Palearctic and only a single species in the Nearctic (*Macrobrachium ohione*).

Three of the other freshwater families, Desmocarididae, Typhlocarididae and Xiphocarididae are monogeneric families comprising of only a handful of species (two, four, two respectively). These families are relatively range restricted, desmocarids to western Africa, typhlocaridids to the circum-Mediterranean and xiphocaridids to the Caribbean. The Euryrhynchidae (Fig. 1C) is a fully freshwater family with three genera, one of which is distributed relatively widely in the northern Neotropics (*Euryrhynchus*), whilst two, monotypic genera (*Euryrhynchina*, *Euryrhynchoides*) are known from western Africa (Afrotropical). The Alpheidae, a primarily marine family containing over 650 species globally, has five freshwater adapted species in the genera *Alpheus* (1 species) and *Potamalpheops* (4 species).

**Fig 3:**
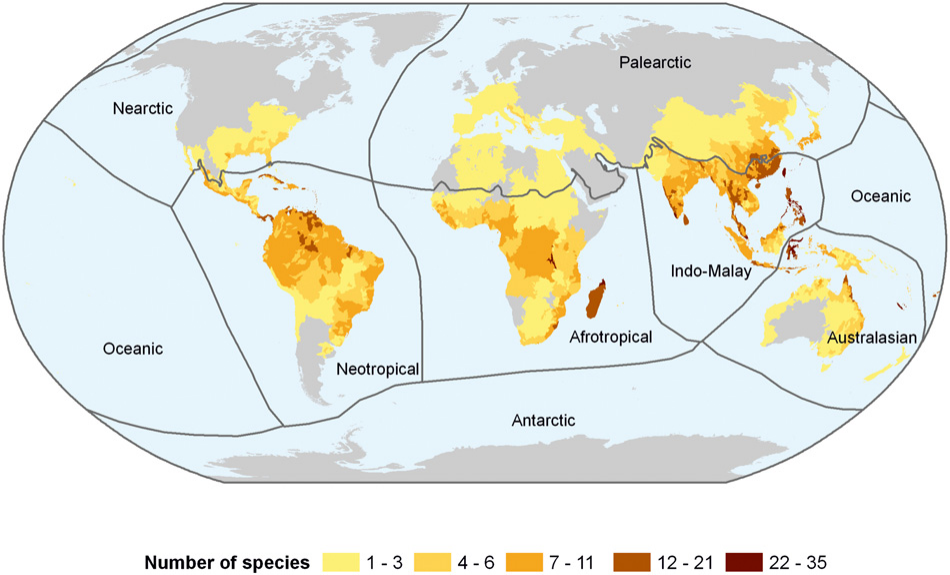
Global species richness of freshwater shrimps, based on 713 out of 763 species.

The Indo-Malayan realm harbours just over half of all known freshwater shrimp species (Table 1), with areas of high species richness in the Philippines, southern China and Taiwan (Fig. 3). The Afrotropical, Neotropical and Australasian realms all host comparatively similar levels of species richness (Table 1) with between 10 and 16% of species, with the ancient lakes of Sulawesi (Australasian realm) representing one of the most speciose localised areas in the world. Madagascar, the Guyana Shield area, and the upper Amazon basin also support high numbers of species. A further notable centre of diversity is New Caledonia (Australasian realm) with 31 species, the highest number for any oceanic island.

**Table 1:** Number of freshwater shrimp species in each threat category, globally and according to biogeographic realm.

	EX	CR	EN	VU	NT	LC	DD	Total	%of all spp.
Global	2	21	38	74	17	329	282	763	
Nearctic	1	1	4	1		7	2	16	2.1
Australasian		1	2	9	1	55	15	83	10.9
Palearctic		1	3	7	5	16	11	43	5.6
Neotropical		5	1	13	2	73	30	124	16.3
Indo-Malayan	1	10	23	40	5	163	174	416	54.5
Afrotropical		2	5	4	3	55	48	117	15.3
Oceania		1			1	27	4	33	4.3

Realms are listed in decreasing order according to total threatened number of taxa (CR + EN + VU, as a percentage of total biodiversity minus EX and DD). Note that a species can be found in more than one realm.

On a country basis (S1 Appendix), China has the highest number of species (159), amounting to 20.8% of the global fauna; followed by Indonesia (116), India (77), Philippines (58), Mexico (48) and Malaysia (48). Within the Afrotropical realm, Madagascar has the highest number of species (40), a number likely to rise as the shrimp fauna remains poorly known [20], as is indeed the case across the entire Afrotropical realm. Australia harbours a similar number of known species (39), which is likely to rise significantly, even though large parts of the country are arid. For example, a significant number of additional *Caridina* species are known, but await formal description. In general, Palearctic countries have a low diversity, with one or two species, but notable exceptions are Croatia and Bosnia/Herzegovina, which hold seven and five species respectively (one containing six subspecies, all assessed as NT), due to localised radiations of the karst dwelling genus, *Troglocaris*.

Nearly two-thirds of freshwater shrimp species are found in flowing water (Table 2), but with a significant component also occurring in both subterranean and permanent lake environments. However, it is the subterranean faunas that are at the greatest risk of extinction with over two-thirds of species assessed as threatened, making up almost half of all threatened freshwater shrimp species.

**Table 2:** Habitats containing over 5% of global species richness of freshwater shrimps (excluding Unknown Habitat) and their status.

Habitat type	Percentage of global species richness in habitat	Percentage threatened within habitat	Percentage of all threatened species in habitat
Permanent Rivers/Streams/Creeks (includes waterfalls)	62.5	12.0	30.8
Karst and Other Subterranean Hydrological Systems	16.4	69.3	45.9
Permanent Freshwater Lakes (over 8ha)	12.7	35.2	23.3
Permanent Freshwater Marshes/Pools (under 8ha)	6.8	7.1	2.3

Note that a species can be assigned to multiple habitats.

**Fig 4:**
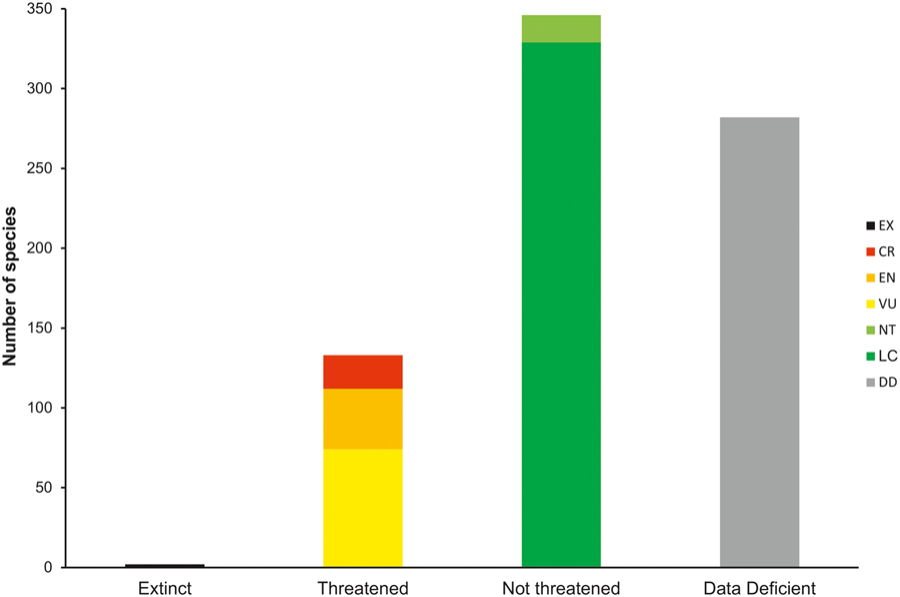
Global extinction risk of freshwater shrimps.

A breakdown of the IUCN threat categories reveals that two species can be considered as EX (Table 1; Fig. 4), with a further 21 species CR, of which 10 are Possibly Extinct (Table 3). Out of the total global diversity of extant freshwater shrimp species for which sufficient information exists to assign a risk of extinction, 27.8% of species are classed as threatened (Fig. 4), with a further 17 species as NT, a situation which could rapidly change given that over one third (37.0%) of freshwater shrimps suffer from a relative paucity of data and are assessed as Data Deficient.

**Table 3:** Freshwater shrimps currently considered as Critically Endangered (Possibly Extinct).

Family	Distribution	Main threats	Last known sighting/specimen collected
**Atyidae**			
*Atya brachyrhinus*	Barbados (single cave)	Ground water extraction; pollution	1970
*Caridina apodosis*	Hong Kong (single stream)	Urbanisation	1994
*Caridina yilong*	Yunnan, China (single lake)	Water abstraction for agriculture	1983
*Sinodina acutipoda*	Yunnan, China (single lake)	Invasive fishes; siltation	1986
**Palaemonidae**			
*Cryphiops luscus*	Chiapas, Mexico (single cave)	Urban waste pollution	1986
*Macrobrachium denticulatum*	Brazil (single river system)	Dams; siltation; sewage pollution	1995
*Macrobrachium oxyphilus*	Malaysia (single peat swamp)	Oil palm conversion	1991
*Macrobrachium purpureamanus*	Indonesia (single peat swamp)	Oil palm conversion	1998
*Macrobrachium scorteccii*	Somalia (single spring)	Drought	1957
*Palaemonetes cummingi*	Florida, USA (single cave)	Pollution; invasive fish	1970s

Their distribution, main threats to the species and their last known sighting is listed.

**Fig 5:**
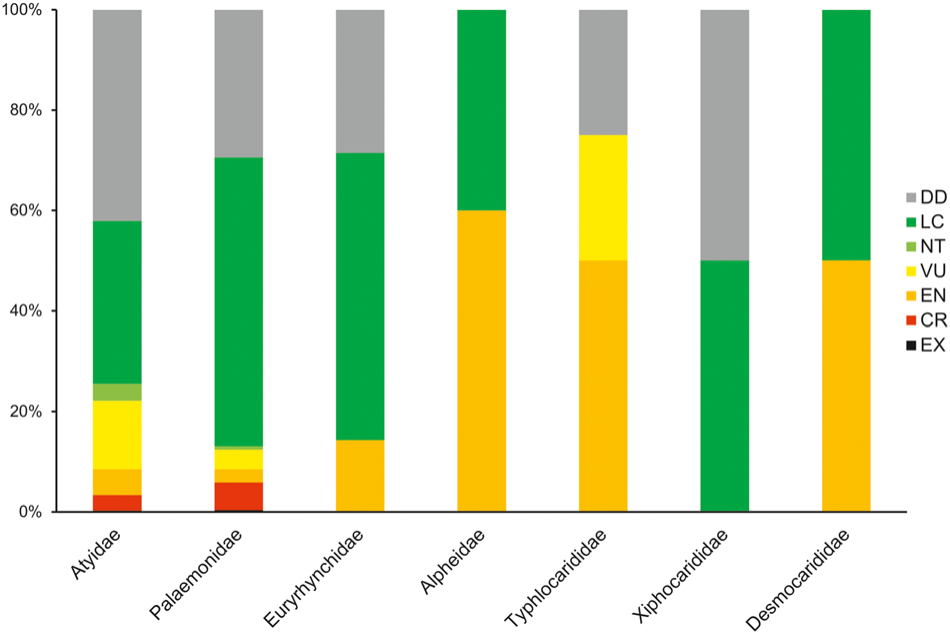
Extinction risk in the various shrimp families, expressed as a percentage of species richness per family.

Different levels of extinction risk are apparent (Fig. 5) at family level, with the extinction risk of Atyidae (37% species threatened) being more than twice that of the other speciose family, Palaemonidae (14.8%). Desmocarididae and Typhlocarididae are highly threatened (50.0 and 100.0% respectively), as are the freshwater members of Alpheidae (60.0%). The lowest extinction risk currently exists in Xiphocarididae at 0%, with one species being LC and one being DD. Noteworthy is the much higher percentage of DD species in Atyidae (42.4%) compared to Palaemonidae (28.6%).

On the basis of biogeographical realms (Table 1), the Nearctic has the highest proportion of threatened species (46.1%), despite harbouring the lowest biodiversity of all realms. The Indo-Malayan region, where over half of global shrimp diversity occurs, has nearly one-third (30.3%) of species threatened, a number which could rise significantly, once the large proportion of DD species can be assigned to a category of extinction risk.

**Fig 6:**
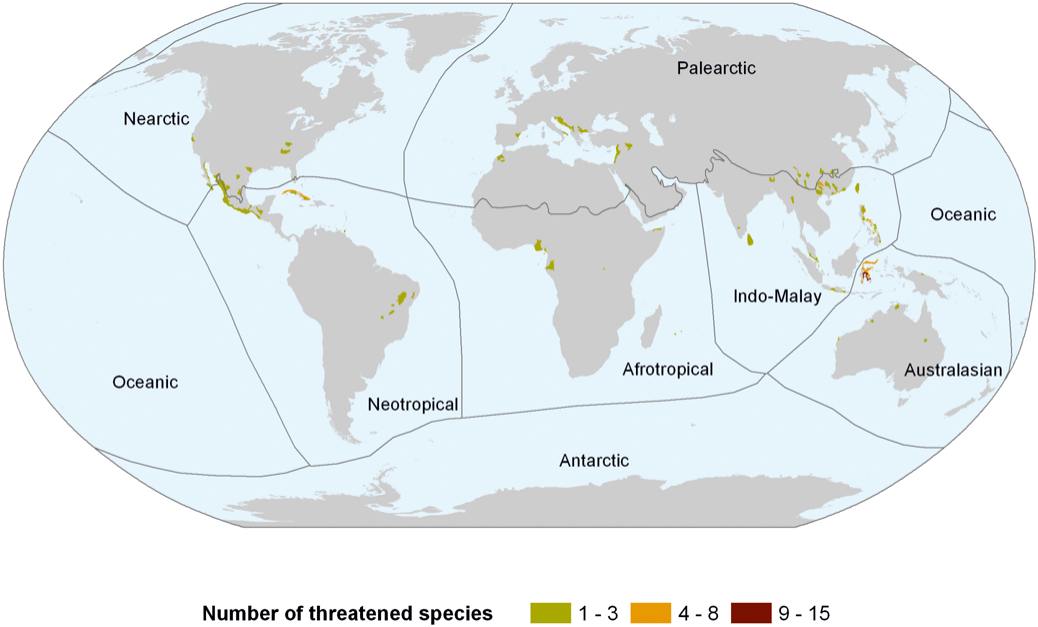
Global distribution of threatened freshwater shrimp species.

Sulawesi in Indonesia has the highest number of threatened species in the world (Fig. 6), followed by Cuba, the Philippines and southern China. Lower numbers of threatened species are scattered across the globe, including Sri Lanka, Australia, Taiwan, the western Balkan Peninsula, central Africa and Mexico.

On a country basis (S1 Appendix), 50% or more of the fauna are threatened in Somalia, Israel, Uganda, Montenegro, Morocco and Syria, admittedly all of which are very species-poor. Of the more species-rich countries, over 40% of the fauna are threatened in Cuba and over 30% in China, Indonesia and the United States. Conversely, the highest proportions of LC species can be found in India, Malaysia, Brazil, Japan and Venezuela, with no species in the latter two countries currently assessed as threatened.

**Fig 7:**
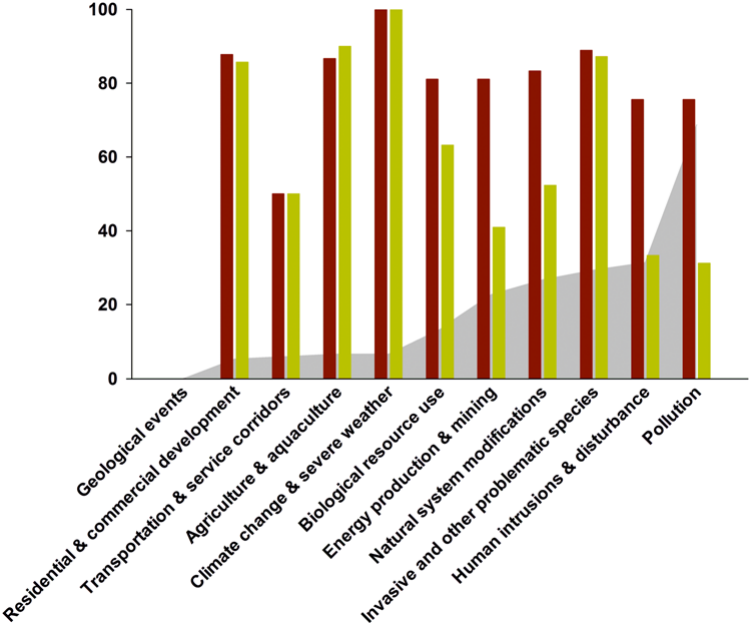
Major threats to freshwater shrimps. Incorporating the proportion of species impacted by each threat, the severity of the threat upon the species, and the overall threat impact score. The proportion of threatened and NT species impacted by each threat category is shown in grey; the proportion of species-threat interactions causing rapid/very rapid declines is shown in red, whilst the average threat impact score (normalised) to 100 is shown in green. Calculations for the latter two indicators are based on threatened and NT species only.

The threats impacting the greatest proportion of threatened and NT species (Fig. 7) are *pollution*, primarily from urban and agricultural sources, which impacts 68.7%; and *human intrusions and disturbance*, which impacts 31.3% (primarily cave dwelling species). *Invasive species* (impacting 29.3%), *natural system modifications* mostly due to dams and water abstraction (26.7%), and *mining* (22.7%) are also significant threats. However, it is *agriculture and aquaculture*, *climate change* and *invasive species* that have the highest overall ‘threat impact score’ (a combination of timing, scope and severity). Of perhaps unique significance amongst freshwater invertebrate groups is the level of wild collecting for the aquarium trade (included under *biological resource use*), with seven threatened or NT species impacted by harvesting for this purpose.

## Discussion

### Global patterns of diversity and threats

Global richness patterns in freshwater shrimps are clearly caused by both localised species radiations and habitat heterogeneity, with their combined effect creating regional diversity peaks. For example, the Western Ghats, Madagascar, the Guyana Shield area, upper Amazon and Indo-China have generally been recognised as having elevated biodiversity levels across many terrestrial and freshwater groups, due to their habitat heterogeneity and, until relatively recently, pristine habitats. Although subterranean shrimp occur worldwide, significantly higher concentrations are found in the karst rich areas in China, the western Balkan Peninsula, the Philippines and Cuba. This richness is underpinned by species radiations, with each cave system harbouring closely related, but unique, species and/or subspecies [21, 22]. Ancient lakes also show high biodiversity peaks, with extensive endemic radiations in Lake Tanganyika [23], and especially in the ancient lake system in Sulawesi [24].

The highest concentration of threatened species is on Sulawesi, where the species flock of *Caridina* is being impacted by harvesting for the aquarium trade due to their attractive colour pattern, whereas the lake fauna in general is impacted by invasive fishes, as well as various sources of pollution [25]. Other concentrations of threatened species are disjunct areas of karst in the Philippines, the high altitude lake region of Yunnan (China), the cave systems in Cuba and large river systems on the Pacific coast of Mexico. In each area pollution plays a significant role, along with mining (Philippines), human intrusions (Cuba) and dams (Mexico).

Two species are currently considered to be extinct. The Californian *Syncaris pasadenae* used to be common before 1900 in streams around Pasadena, now part of the Los Angeles conurbation, but despite extensive surveys, the species has not been found since 1933 [26]. *Macrobrachium leptodactylus* is only known from material collected in 1888 in Java and despite semi-annual surveys, since the early 1990s, has not been encountered again. However, the taxonomic status of this species has been questioned, and it could be a synonym of a widespread, LC species in the *M*. *pilimanus* complex. A further ten point-endemic species are currently considered as CR, but in all likelihood are also extinct as recent surveys in their known localities have failed to find them (Table 3). Since these surveys have not been rigorous enough to meet IUCN criteria to classify the species as EX, their status remains as CR (Possibly Extinct) for now.

Few freshwater invertebrate groups have been globally assessed. However, shrimps with 27.8% threatened species are comparable with crabs (32.0%), and crayfishes (31.5%) [27, 28], which can be explained by similar threats and habitats. The slightly lower percentage is likely caused by many freshwater shrimp species having marine pelagic larvae [29], allowing for a larger distributional range than freshwater crabs and crayfishes, which have direct development. Differences in larval development also explain the different threat levels between the two, dominant shrimp families. Proportionally more Atyidae are land-locked and/or have abbreviated larval development, resulting in more restricted ranges, often a single cave or lake and thus are more subject to pollution and other threats.

### Threat analysis

Not all threats impact biodiversity to the same degree and identifying which threats are ‘major’ and which are ‘minor’ depends upon a number of aspects, such as timing, scope and severity, which can be combined into threat impact scores (see Methods section and Fig. 7).

While pollution impacts over two-thirds (69%) of all threatened and NT shrimp species (over twice the number as any other threat), it has the second lowest average threat impact score, and the lowest threat *severity* (with 31% species undergoing *very rapid* or *rapid* declines). This means that while pollution impacts a large number of species it is less likely to have a severe impact upon shrimp populations than the other threats. The threat categories with the highest average threat impact scores are climate change, invasive species and residential and commercial development. Of these, invasive species impact the highest proportion of threatened and NT shrimp species (29%), and potentially causes 88.9% of these species to undergo *very rapid* or *rapid* declines (the second highest severity of all the threats). Examples of these impacts are: introduced guppies and trout predating upon juveniles of *Lancaris kumariae* (CR), which is known only from a 3–4 km stretch of a stream in Sri Lanka [30]; increased urbanisation around Valencia in Spain, impacting the range restricted *Dugastella valentina* (NT) [31]; and climate change impacting several cave dwelling taxa (*Stygiocaris* spp., *Typhlocaridina* spp.) by lowering ground water level due to prolonged droughts [32].

### Data deficiency

The overall level of DD species (where there is inadequate information to assess risk of extinction) presently stands at 282 species (37.0% of all species), a high proportion of which are not known beyond their original, type descriptions. This is not an unusual level of deficiency when assessing invertebrate taxa, with for instance 49.1% in freshwater crabs [27] and 21.0% in crayfishes [28]. This is particularly acute in China, the most species-rich country for freshwater shrimps, with 159 species (37.5% threatened), 95 of which are DD (59.7% of all Chinese species). Broadly speaking, this is similar to the number of DD freshwater crabs in China, which stands at 77.7% [27]. Taxonomic uncertainty certainly accounts for some of this deficiency, however many of these species are not known beyond their type locality and their actual geographic ranges are unclear due to inadequate on-the-ground knowledge. Xie and Chen [33] already drew attention to large scale deterioration of freshwater habitats in China and the resulting severe declines in fish diversity, a situation which is unlikely to have improved with China’s economic growth in the last decade. It therefore seems highly likely that the true number of threatened freshwater shrimp in China (and probably other countries) is greater than herein estimated.

A similar situation exists for the whole of the Afrotropical region, where 41.0% are considered to be DD. For many of these species, no accounts are available beyond their original descriptions, often based on specimens collected pre-1950 and without much information available as to their current distributions, population sizes or potential threats, similar to the freshwater crab fauna [27].

### Conservation and research recommendations

Freshwater shrimps on the whole do not differ in their habitat requirements from a broad range of other freshwater taxa. As such many of the recommendations for habitat and species conservation apply. These are primarily the incorporation of the needs of biodiversity within water management decision making processes through the adoption of integrated water resource management (IWRM) principles, environmental flow concepts and comprehensive environmental and social impact assessments (EISAs). A better recognition of the needs of freshwater biodiversity within protected area management plans [34, 35] is also required.

Of particular relevance, and unique within freshwater invertebrates, is the need for brackish or fully marine water for larval development [29] in 14% of all species (108 species) included in the current assessment. However, this percentage is likely a significant underestimate as for numerous species reproductive biology and life cycles are poorly or not known. Nevertheless, the impact of dams and weirs potentially blocking migratory routes of catadromous shrimp may be highly significant. Although anecdotal evidence supports the notion that dams and weirs have significantly impacted the distribution of several shrimp species, notably some Mexican and South African Palaemonidae, potential mitigating measures have been poorly studied so far, in contrast to fish and needs to be put on the research agenda.

A significant proportion of the fauna also inhabits karst and other subterranean environments, with numerous species having highly localised distributions and being under multiple threats, from groundwater extraction and climate change to pollution of surface catchments. More research is urgently required into the ecologies of the species concerned, whilst legislative actions possibly including site level protection needs to be taken to protect this globally fragile ecosystem.

## Supporting Information

S1 Appendix Alphabetical list of countries and territories, harbouring freshwater shrimps, showing: (1) total species richness, (2) numbers of species in each threat category (see text for details), and (3) overall percentage of threatened species. Country and territory names follow ISO 3166.
(DOCX)
