# Supplementary material for: Dead Shrimp Blues: A Global Assessment of Extinction Risk in Freshwater Shrimps (Crustacea: Decapoda: Caridea)
Source: PLoS One. 2015 Mar 25;10(3):e0120198. doi: 10.1371/journal.pone.0120198 (PMC4373683; doi:10.1371/journal.pone.0120198)
Supplement: S1 Appendix — Country and territory names follow ISO 3166. (DOCX) [file pone.0120198.s001.docx]

**Appendix S1.**

**Alphabetical list of countries and territories, harbouring freshwater shrimps, showing: (1) total species richness, (2) numbers of species in each threat category (see text for details), and (3) overall percentage of threatened species.** Country and territory names follow ISO 3166.

|  | **Total species richness** | **EX** | **CR** | **EN** | **VU** | **NT** | **LC** | **DD** | **% threatened in country** |
| --- | --- | --- | --- | --- | --- | --- | --- | --- | --- |
| Algeria | 2 |  |  |  |  |  | 1 | 1 | 0.00 |
| Angola | 6 |  |  |  |  |  | 4 | 2 | 0.00 |
| Antigua and Barbuda | 2 |  |  |  |  |  | 2 |  | 0.00 |
| Argentina | 5 |  |  |  |  |  | 5 |  | 0.00 |
| Australia | 39 |  | 1 | 1 | 6 | 1 | 28 | 2 | 21.62 |
| Austria | 1 |  |  |  |  |  | 1 |  | 0.00 |
| Bangladesh | 9 |  |  |  |  |  | 8 | 1 | 0.00 |
| Barbados | 7 |  | 1 |  |  |  | 6 |  | 14.29 |
| Belgium | 1 |  |  |  |  |  | 1 |  | 0.00 |
| Belize | 1 |  |  |  |  |  | 1 |  | 0.00 |
| Benin | 5 |  |  |  |  |  | 5 |  | 0.00 |
| Bolivia, Plurinational States of | 6 |  |  |  |  |  | 6 |  | 0.00 |
| Bonaire, Sint Eustatius and Saba | 2 |  |  |  |  |  | 2 |  | 0.00 |
| Bosnia and Herzegovina | 5 |  |  |  | 2 | 2 | 1 |  | 40.00 |
| Botswana | 1 |  |  |  |  |  | 1 |  | 0.00 |
| Brazil | 35 |  | 2 |  |  |  | 33 |  | 5.71 |
| Brunei Darussalam | 9 |  |  |  |  |  | 9 |  | 0.00 |
| Burkina Faso | 1 |  |  |  |  |  | 1 |  | 0.00 |
| Burundi | 10 |  |  |  |  |  | 10 |  | 0.00 |
| Cambodia | 3 |  |  |  |  |  | 2 | 1 | 0.00 |
| Cameroon | 13 |  |  |  | 1 |  | 10 | 2 | 9.09 |
| Cape Verde | 3 |  |  |  |  |  | 3 |  | 0.00 |
| Central African Republic | 1 |  |  |  |  |  | 1 |  | 0.00 |
| Chad | 2 |  |  |  |  |  | 2 |  | 0.00 |
| Chile | 1 |  |  |  |  |  | 1 |  | 0.00 |
| China | 159 |  | 3 | 2 | 19 | 1 | 39 | 95 | 37.50 |
| Christmas Island | 1 |  |  |  |  |  | 1 |  | 0.00 |
| Colombia | 30 |  |  |  |  |  | 26 | 4 | 0.00 |
| Comoros | 2 |  |  |  |  |  | 2 |  | 0.00 |
| Congo | 8 |  |  |  |  |  | 6 | 2 | 0.00 |
| Congo, DR | 26 |  |  |  |  |  | 21 | 5 | 0.00 |
| Costa Rica | 16 |  |  |  | 1 | 1 | 13 | 1 | 6.67 |
| Côte d'Ivoire | 7 |  |  |  |  |  | 4 | 3 | 0.00 |
| Croatia | 7 |  |  |  | 2 | 2 | 3 |  | 28.57 |
| Cuba | 21 |  |  |  | 8 | 1 | 10 | 2 | 42.11 |
| Curaçao | 7 |  |  |  |  |  | 7 |  | 0.00 |
| Czech Republic | 1 |  |  |  |  |  | 1 |  | 0.00 |
| Dominica | 8 |  |  |  |  |  | 8 |  | 0.00 |
| Dominican Republic | 8 |  |  |  |  |  | 8 |  | 0.00 |
| Ecuador | 10 |  |  |  |  |  | 9 | 1 | 0.00 |
| Egypt | 3 |  |  |  |  |  | 3 |  | 0.00 |
| El Salvador | 7 |  |  |  |  | 1 | 6 |  | 0.00 |
| Equatorial Guinea | 8 |  |  | 1 |  |  | 6 | 1 | 14.29 |
| Fiji | 19 |  |  |  |  |  | 16 | 3 | 0.00 |
| France | 2 |  |  |  |  | 1 | 1 |  | 0.00 |
| French Guiana | 7 |  |  |  |  |  | 6 | 1 | 0.00 |
| French Polynesia | 10 |  |  |  |  |  | 10 |  | 0.00 |
| Gabon | 11 |  |  | 1 |  |  | 8 | 2 | 11.11 |
| Georgia | 5 |  |  |  |  |  |  | 5 | N/A |
| Germany | 1 |  |  |  |  |  | 1 |  | 0.00 |
| Ghana | 8 |  |  |  |  |  | 5 | 3 | 0.00 |
| Greece | 4 |  |  |  | 1 |  | 3 |  | 25.00 |
| Grenada | 5 |  |  |  |  |  | 5 |  | 0.00 |
| Guadeloupe | 7 |  |  |  |  |  | 7 |  | 0.00 |
| Guam | 4 |  |  |  |  |  | 4 |  | 0.00 |
| Guatemala | 8 |  |  |  |  | 1 | 7 |  | 0.00 |
| Guinea | 8 |  |  |  |  |  | 7 | 1 | 0.00 |
| Guyana | 9 |  |  |  |  |  | 8 | 1 | 0.00 |
| Haiti | 6 |  |  |  |  |  | 6 |  | 0.00 |
| Holy See (Vatican City State) | 2 |  |  |  |  |  | 2 |  | 0.00 |
| Honduras | 5 |  |  |  |  |  | 4 | 1 | 0.00 |
| Hong Kong | 5 |  | 1 |  | 1 | 1 | 2 |  | 40.00 |
| India | 77 |  |  | 1 | 1 |  | 46 | 29 | 4.17 |
| Indonesia | 116 | 1 | 2 | 17 | 10 |  | 66 | 20 | 30.53 |
| Iran | 4 |  |  |  |  |  | 4 |  | 0.00 |
| Iraq | 3 |  |  |  |  |  | 3 |  | 0.00 |
| Israel | 3 |  |  | 2 |  |  | 1 |  | 66.67 |
| Italy | 4 |  |  |  | 1 |  | 3 |  | 25.00 |
| Jamaica | 12 |  |  |  |  |  | 11 | 1 | 0.00 |
| Japan | 33 |  |  |  |  |  | 30 | 3 | 0.00 |
| Jordan | 1 |  |  |  |  |  | 1 |  | 0.00 |
| Kazakhstan | 1 |  |  |  |  |  | 1 |  | 0.00 |
| Kenya | 12 |  |  |  |  |  | 10 | 2 | 0.00 |
| Korea, DPR of | 2 |  |  |  |  |  | 1 | 1 | 0.00 |
| Korea, Republic of | 5 |  |  |  |  |  | 3 | 2 | 0.00 |
| Lao People's Democratic Republic | 11 |  |  |  |  |  | 11 |  | 0.00 |
| Lebanon | 2 |  |  |  |  |  | 1 | 1 | 0.00 |
| Liberia | 9 |  |  |  |  |  | 9 |  | 0.00 |
| Libya | 1 |  |  |  |  |  |  | 1 | N/A |
| Macedonia | 1 |  |  |  |  |  | 1 |  | 0.00 |
| Madagascar | 40 |  |  |  |  |  | 20 | 20 | 0.00 |
| Malawi | 3 |  |  |  |  |  | 1 | 2 | 0.00 |
| Malaysia | 48 |  | 1 | 2 |  |  | 44 | 1 | 6.38 |
| Mali | 2 |  |  |  |  |  | 2 |  | 0.00 |
| Martinique | 4 |  |  |  |  |  | 4 |  | 0.00 |
| Mauritius | 7 |  |  | 1 |  | 3 | 3 |  | 14.29 |
| Mexico | 48 |  | 3 | 1 | 4 | 1 | 29 | 10 | 21.05 |
| Montenegro | 2 |  |  |  | 1 | 1 |  |  | 50.00 |
| Montserrat | 2 |  |  |  |  |  | 2 |  | 0.00 |
| Morocco | 2 |  |  | 1 |  |  | 1 |  | 50.00 |
| Mozambique | 7 |  |  |  |  |  | 7 |  | 0.00 |
| Myanmar | 23 |  |  | 2 |  |  | 17 | 4 | 10.53 |
| Nepal | 5 |  |  |  |  |  | 4 | 1 | 0.00 |
| Netherlands | 1 |  |  |  |  |  | 1 |  | 0.00 |
| New Caledonia | 20 |  |  |  |  |  | 19 | 1 | 0.00 |
| New Zealand | 1 |  |  |  |  |  | 1 |  | 0.00 |
| Nicaragua | 9 |  |  |  |  |  | 9 |  | 0.00 |
| Nigeria | 13 |  |  | 3 |  |  | 9 | 1 | 25.00 |
| Norfolk Island | 1 |  | 1 |  |  |  |  |  | 100.00 |
| Northern Mariana Islands | 2 |  |  |  |  |  | 2 |  | 0.00 |
| Pakistan | 3 |  |  |  |  |  | 3 |  | 0.00 |
| Palau | 3 |  |  |  |  |  | 3 |  | 0.00 |
| Panama | 25 |  |  |  | 1 | 1 | 20 | 3 | 4.55 |
| Papua New Guinea | 23 |  |  |  |  |  | 16 | 7 | 0.00 |
| Paraguay | 2 |  |  |  |  |  | 2 |  | 0.00 |
| Peru | 15 |  |  |  |  |  | 11 | 4 | 0.00 |
| Philippines | 58 |  | 1 | 1 | 12 |  | 36 | 8 | 28.00 |
| Poland | 1 |  |  |  |  |  | 1 |  | 0.00 |
| Portugal | 1 |  |  |  |  |  | 1 |  | 0.00 |
| Puerto Rico | 11 |  |  |  |  |  | 11 |  | 0.00 |
| Réunion | 6 |  |  | 1 |  |  | 5 |  | 16.67 |
| Russian Federation | 3 |  |  |  |  |  | 2 | 1 | 0.00 |
| Saint Kitts and Nevis | 1 |  |  |  |  |  | 1 |  | 0.00 |
| Saint Lucia | 3 |  |  |  |  |  | 3 |  | 0.00 |
| Saint Vincent and the Grenadines | 3 |  |  |  |  |  | 3 |  | 0.00 |
| Samoa | 4 |  |  |  |  |  | 4 |  | 0.00 |
| São Tomé and Principe | 5 |  |  | 1 |  |  | 3 | 1 | 25.00 |
| Senegal | 4 |  |  |  |  |  | 4 |  | 0.00 |
| Seychelles | 5 |  |  |  |  |  | 5 |  | 0.00 |
| Sierra Leone | 6 |  |  |  |  |  | 5 | 1 | 0.00 |
| Singapore | 21 |  |  | 1 |  |  | 20 |  | 4.76 |
| Slovenia | 2 |  |  |  |  | 1 | 1 |  | 0.00 |
| Solomon Islands | 1 |  |  |  |  |  | 1 |  | 0.00 |
| Somalia | 2 |  | 1 |  |  |  |  | 1 | 100.00 |
| South Africa | 13 |  |  |  |  |  | 9 | 4 | 0.00 |
| Spain | 3 |  |  |  | 1 | 1 | 1 |  | 33.33 |
| Sri Lanka | 17 |  | 1 |  |  | 1 | 13 | 2 | 6.67 |
| Sudan | 2 |  |  |  |  |  | 2 |  | 0.00 |
| Suriname | 11 |  |  |  |  |  | 11 |  | 0.00 |
| Syrian Arab Republic | 3 |  | 1 |  |  |  | 1 | 1 | 50.00 |
| Taiwan | 28 |  |  |  | 1 | 2 | 21 | 4 | 4.17 |
| Tanzania, United Republic of | 19 |  |  |  |  |  | 18 | 1 | 0.00 |
| Thailand | 31 |  |  |  |  |  | 29 | 2 | 0.00 |
| Togo | 4 |  |  |  |  |  | 2 | 2 | 0.00 |
| Tonga | 1 |  |  |  |  |  | 1 |  | 0.00 |
| Trinidad and Tobago | 11 |  |  |  |  |  | 11 |  | 0.00 |
| Tunisia | 2 |  |  |  |  |  | 1 | 1 | 0.00 |
| Turkey | 5 |  | 1 |  |  |  | 3 | 1 | 25.00 |
| Turks and Caicos Islands | 1 |  |  |  | 1 |  |  |  | 100.00 |
| Uganda | 7 |  | 1 |  | 3 |  | 2 | 1 | 66.67 |
| United States | 21 | 1 | 1 | 4 | 1 | 1 | 12 | 1 | 31.58 |
| Uruguay | 3 |  |  |  |  |  | 3 |  | 0.00 |
| Uzbekistan | 1 |  |  |  |  |  | 1 |  | 0.00 |
| Vanuatu | 5 |  |  |  |  |  | 4 | 1 | 0.00 |
| Venezuela, Bolivarian Republic of | 36 |  |  |  |  |  | 30 | 6 | 0.00 |
| Viet Nam | 32 |  |  |  |  |  | 17 | 15 | 0.00 |
| Virgin Islands, U.S. | 5 |  |  |  |  |  | 5 |  | 0.00 |
| Zambia | 17 |  |  |  |  |  | 16 | 1 | 0.00 |
| Zimbabwe | 2 |  |  |  |  |  | 2 |  | 0.00 |
